# A Novel Noncircular MUSIC Algorithm Based on the Concept of the Difference and Sum Coarray

**DOI:** 10.3390/s18020344

**Published:** 2018-01-25

**Authors:** Zhenhong Chen, Yingtao Ding, Shiwei Ren, Zhiming Chen

**Affiliations:** School of Information and Electronics, Beijing Institute of Technology, 5 South Zhongguancun Street, Haidian District, Beijing 100081, China; chenzhenhongbit@163.com (Z.C.); ytd@bit.edu.cn (Y.D.); renshiwei@bit.edu.cn (S.R.)

**Keywords:** noncircular signals, DOA estimation, virtual array, coprime array

## Abstract

In this paper, we propose a vectorized noncircular MUSIC (VNCM) algorithm based on the concept of the coarray, which can construct the difference and sum (diff–sum) coarray, for direction finding of the noncircular (NC) quasi-stationary sources. Utilizing both the NC property and the concept of the Khatri–Rao product, the proposed method can be applied to not only the ULA but also sparse arrays. In addition, we utilize the quasi-stationary characteristic instead of the spatial smoothing method to solve the coherent issue generated by the Khatri–Rao product operation so that the available degree of freedom (DOF) of the constructed virtual array will not be reduced by half. Compared with the traditional NC virtual array obtained in the NC MUSIC method, the diff–sum coarray achieves a higher number of DOFs as it comprises both the difference set and the sum set. Due to the complementarity between the difference set and the sum set for the coprime array, we choose the coprime array with multiperiod subarrays (CAMpS) as the array model and summarize the properties of the corresponding diff–sum coarray. Furthermore, we develop a diff–sum coprime array with multiperiod subarrays (DsCAMpS) whose diff–sum coarray has a higher DOF. Simulation results validate the effectiveness of the proposed method and the high DOF of the diff–sum coarray.

## 1. Introduction

Noncircular (NC) signals, such as amplitude-modulated (AM) signals and binary phase-shift keying (BPSK)-modulated signals, have been widely applied in various communication systems [1,2,3,4,5,6,7,8]. Different from circular signals, which can only use the information in the covariance matrix for direction finding, NC signals can use the information in both the covariance matrix and the elliptic covariance matrix for direction finding. This NC property can be utilized to increase the degrees of freedom (DOFs) and improve the estimation performance. A lot of DOA estimation algorithms for NC sources have been developed, such as the NC MUSIC method [9], NC Root-MUSIC method [1], NC ESPRIT method [10] and NC Unitary ESPRIT method [11]. These traditional NC DOA estimation algorithms utilize the complex conjugate counterpart of the received signals to obtain the NC covariance matrix, which corresponds to a virtual array consisting of the physical array and its flipped array [12]. These algorithms mostly utilize the uniform linear array (ULA) as the array model and can detect at most 2(N−1) sources with *N* physical sensors. In order to detect more sources, some NC high-order cumulant MUSIC methods based on the non-Gaussian characteristic of many NC sources, such as the NC 2q-MUSIC method [13] and ROOT NC 4-MUSIC method [2], have been proposed. However, the array model in these methods is still the ULA, and the computation complexity of the cumulant-based methods are large. As we know, the array aperture is a fundamental parameter that affects the performance of the DOA estimation. For the ULA, its aperture is usually less than that of the sparse array constructed based on the concept of the coarray [14,15,16,17,18,19]. Thus, an estimator, which jointly utilizes the NC property, the sparse array and the concept of the coarray, is likely to detect more sources than the traditional NC DOA estimators.

In recent years, sparse arrays have been attracting more and more attention due to the high number of DOFs [20,21,22,23]. The concept of the coarray based on the Khatri–Rao (KR) product [21,24] is applied to design various of sparse arrays, such as the coprime array [22]. The coprime array consists of two uniform linear subarrays with *M* and *N* sensors, respectively, where *M* and *N* are a coprime pair of positive integers. The *M* element subarray with spacing *N* units and the *N* element subarray with spacing *M* units share the first sensor. By applying the KR product to the covariance matrix of the received signals, the difference coarray [21,22,23,24,25,26,27] of the coprime array is constructed. With only M+N−1 physical sensors, the virtual array can detect O(MN) sources. In order to achieve a higher number of DOFs, many novel methods and optimized configurations based on the difference coarray for the coprime array have been proposed, such as the sparsity enforced recovery technique for the coprime array [18], the coarray interpolation method for the coprime array [28], the extended coprime array [29], the generalized coprime arrays [24] and the coprime array with multiperiod subarrays (CAMpS) [30,31]. Since these methods and improved configurations are developed based on the properties of the difference coarray for the coprime array, their DOFs cannot be more than twice the physical aperture. Constructing a novel virtual array with larger array aperture than the difference coarray is another useful way to increase the DOFs. In [16], we utilized both the temporal information and the spatial information of the received signals to propose the vectorized conjugate augmented MUSIC (VCAM) algorithm, which can construct the difference and sum (diff–sum) coarray. The diff–sum coarray comprises both the difference set and the sum set so that it can achieve a higher number of DOFs than the above difference coarrays. In addition, the aperture of the diff–sum coarray can be more than twice the physical aperture, which could help to decrease the array size. However, the source signals applied in both the VCAM algorithm and the methods constructing the difference coarray are circular signals.

In this paper, we propose an improved NC MUSIC algorithm based on the concept of the coarray to perform the DOA estimation of NC quasi-stationary sources. We name the novel method as the vectorized NC MUSIC (VNCM) method. By applying the KR product operation to the NC covariance matrix, we can obtain an equivalent received signal, which seems to be received from a diff–sum coarray. The diff–sum coarray, which is symmetrical with the zero point as the center, can be divided into three parts: the difference coarray, the nonpositive sum coarray and the non-negative sum coarray. Thus, the diff–sum coarray is likely to achieve a higher number of DOFs than the traditional NC virtual array consisting of the physical array and its flipped array. Also, we utilize the characteristic of the quasi-stationary sources instead of the spatial smoothing method to solve the single snapshot issue (the coherent issue) of the equivalent received signal so that the available DOFs will not be reduced by half. Due to the complementarity between the difference set and the sum set for the coprime array [16], we utilize the CAMpS, which contains the prototype coprime array as a special case, as the basic array model in this paper. In particular, we summarize and prove the properties and DOFs of the diff–sum coarray for the CAMpS. Furthermore, we improve the CAMpS to propose a diff–sum coprime array with multiperiod subarrays (DsCAMpS) which can achieve a higher number of DOFs than the CAMpS. Extensive simulations are provided to verify the performance of the diff–sum coarray and the effectiveness of the VNCM method.

Notations: Throughout the paper, we utilize lowercase bold italic letters to denote vectors, for example, a. We utilize capital bold italic letters to denote matrices, for example, A. ${\left(.\right)}^{T}$, ${\left(.\right)}^{*}$ and ${\left(.\right)}^{H}$ respectively represent transpose, conjugation and conjugate transpose. $E\left[\cdot \right]$ is used to denote the expectation operation and vec(.) represents the vectorizing operation. ⊗ and ⊙ respectively denote the left Kronecker product and the Khatri–Rao product.

## 2. Data Model

Consider a linear antenna array consisting of *R* elements. The set of the sensor locations is denoted as $d=\{d_{1},\ldots ,d_{R}\}$. The first sensor is selected as the reference, that is, $d_{1}=0$. *Q* far-field, narrowband, uncorrelated and NC sources impinge on this array from directions $\left\{\theta_{1},\ldots ,\theta_{Q}\right\}$. The NC sources in the paper are assumed as wide-sense quasi-stationary [32,33] with the NC rate as ρ=1 [13,34]. Then, the received signal can be modeled as

(1) $$\begin{array}{c} x\left(t\right)=\sum_{q=1}^{Q}a(\theta_{q})s_{q}\left(t\right)+n\left(t\right)=\mathrm{As}\left(t\right)+n\left(t\right), \end{array}$$

where $s\left(t\right)={\left[s_{1}\left(t\right),s_{2}\left(t\right),\ldots ,s_{Q}\left(t\right)\right]}^{T}$ is the observed signal vector, $n\left(t\right)={\left[n_{1}\left(t\right),n_{2}\left(t\right),\ldots ,n_{R}\left(t\right)\right]}^{T}$ represents the white circular complex Gaussian noise vector with zero mean and variance $\sigma_{n}^{2}$, and $A=[a\left(\theta_{1}\right),a\left(\theta_{2}\right),\ldots ,a\left(\theta_{Q}\right)]$ is the steering matrix. The *q*th (q=1,2,…,Q) column vector of A is $a\left(\theta_{q}\right)={\left[1,e^{j2\pi d_{2}\sin\left(\theta_{q}\right)/\lambda },\ldots ,e^{j2\pi d_{R}\sin\left(\theta_{q}\right)/\lambda }\right]}^{T}$ with λ being the signal wavelength.

According to the noncircularity of the signals, the following relationships exist:

(2) $$\begin{array}{c} s_{q}\left(t\right)=e^{j\varphi_{q}/2\;}{s_{q}}^{\prime }\left(t\right),q=1,2,\ldots ,Q \end{array}$$

and

(3) $$\begin{array}{c} E\left[s_{q}^{2}\left(t\right)\right]=e^{j\varphi_{q}}E\left[s_{q}\left(t\right)s_{q}^{*}\left(t\right)\right], \end{array}$$

where $\varphi_{q}$ is the NC phase of $s_{q}\left(t\right)$, ${s_{q}}^{\prime }\left(t\right)$ is real-valued with zero-phase, $E[s_{q}\left(t\right)s_{q}^{*}\left(t\right)]$ is the covariance and $E[s_{q}^{2}\left(t\right)]$ is the eliptic covariance. As the signals are quasi-stationary, the following assumptions [33] hold as well.

(A1) Each signal $s_{q}\left(t\right)$ is wide-sense quasi-stationary with the frame length being *L* and the total number of frames being $\tilde{L}$, that is,

(4) $$\begin{array}{c} E\left[{\left|s_{q}\langle i\rangle \right|}^{2}\right]\approx \sum_{t=(i-1)L}^{iL-1}s_{q}\left(t\right)s_{q}^{*}\left(t\right)/L\;\approx \sigma_{q}^{2}\left[i\right],i=1,2,\ldots ,\tilde{L}, \end{array}$$

where $s_{q}\langle i\rangle =s_{q}\left(t\right),t=\left(i-1\right)L,\left(i-1\right)L+1\ldots ,iL-1$ represents the snapshots of the *q*th signal in the *i*th frame.

(A2) The variance sequence $\sigma_{q}^{2}\left[i\right],i=1,\ldots ,\tilde{L}$ is wide-sense stationary and uncorrelated with each other. That is to say, $E\left[\sigma_{q}^{2}\left[i\right]\right]={\bar{m}}_{q}$, $E\left[{\left(\sigma_{q}^{2}\left[i\right]-{\bar{m}}_{q}\right)}^{2}\right]={\tilde{\sigma }}_{q}^{2}$ and $E\left[\left(\sigma_{q_{1}}^{2}\left[i\right]-{\bar{m}}_{q_{1}}\right)\left(\sigma_{q_{2}}^{2}\left[i\right]-{\bar{m}}_{q_{2}}\right)\right]=0$, $q_{1}\neq q_{2}$.

For the *i*th $\left(i=1,\ldots ,\tilde{L}\right)$ frame, combining the received signal and its conjugate version together results in the following NC observation vector,

(5) $$\begin{array}{c} r\left(t\right)=\left[\begin{array}{c} x(t)\\ x^{*}\left(t\right) \end{array}\right]=\left[\begin{array}{c} A\Gamma \\ {\left(A\Gamma \right)}^{*} \end{array}\right]s^{\prime }\left(t\right)+\left[\begin{array}{c} n(t)\\ n^{*}\left(t\right) \end{array}\right]=A^{\prime }s^{\prime }\left(t\right)+n^{\prime }\left(t\right), \end{array}$$

where $\Gamma =diag(e^{j\varphi_{1}/2\;},e^{j\varphi_{2}/2\;},\ldots ,e^{j\varphi_{Q}/2\;})$, $s^{\prime }\left(t\right)={\left[{s^{\prime }}_{1}\left(t\right),{s^{\prime }}_{2}\left(t\right),\ldots ,{s^{\prime }}_{Q}\left(t\right)\right]}^{T}$, $A^{\prime }={\left[{\left(A\Gamma \right)}^{T},{\left(A\Gamma \right)}^{H}\right]}^{T}$ and $n^{\prime }\left(t\right)={\left[n^{T}\left(t\right),n^{H}\left(t\right)\right]}^{T}$. The *q*th column vector of $A^{\prime }$ is $a^{\prime }\left(\theta_{q}\right)={\left[e^{j\varphi_{q}/2\;}a^{T}\left(\theta_{q}\right),e^{-j\varphi_{q}/2\;}a^{H}\left(\theta_{q}\right)\right]}^{T}$, which can be considered to correspond to a traditional NC virtual array consisting of 2R−1 different virtual sensors with the zero point as the center. From (5), the covariance matrix of r(t) can be obtained as

(6) $$\begin{array}{c} \begin{array}{c} R_{rr}\left[i\right]=E\left[r\left(t\right)r^{H}\left(t\right)\right]=A_{NC}R_{s^{\prime }s^{\prime }}A_{NC}^{H}+\sigma_{n}^{2}I, \end{array} \end{array}$$

where $R_{s^{\prime }s^{\prime }}=diag\left(\sigma_{1}^{2}\left[i\right],\sigma_{2}^{2}\left[i\right],\ldots ,\sigma_{Q}^{2}\left[i\right]\right)$ and $A_{NC}={\left[A^{T},{\left(A\phi \right)}^{H}\right]}^{T}=\left[a_{NC}\left(\theta_{1}\right),a_{NC}\left(\theta_{2}\right),\ldots ,a_{NC}\left(\theta_{Q}\right)\right]$ with $a_{NC}\left(\theta_{q}\right)={\left[a^{T}\left(\theta_{q}\right),e^{-j\varphi_{q}}a^{H}\left(\theta_{q}\right)\right]}^{T}$ and $\phi =diag(e^{j\varphi_{1}},e^{j\varphi_{2}},\ldots ,e^{j\varphi_{Q}})$. The traditional NC DOA estimators, of which the array model is the ULA, directly perform eigenvalue decomposition on $R_{rr}\left[i\right]$ to obtain the noise subspace $U_{n}$. According to [1,9], the maximum number of detectable signals (MNDS) is determined by the degree of $\det\{a_{blk}^{H}U_{n}U_{n}^{H}a_{blk}\}$ with $a_{blk}=blkdiag\left(a\left(\theta_{q}\right),a^{*}\left(\theta_{q}\right)\right)$. Therefore, at most 2R−2 sources can be detected. Since the DOF of the traditional NC virtual array with *R* sensors is 2R−1, we have MNDS = DOF−1.

## 3. Vectorized NC MUSIC Algorithm

In this paper, we focus on combining the NC property and the concept of the coarray. NC DOA estimators based on the concept of the coarray have two problems to be resolved: (1) how to solve the single snapshot issue (the coherent issue) of the equivalent received signal obtained by the KR product operation; and (2) how to generate a coarray with a large array aperture. In order to resolve the two problems, we propose a vectorized NC MUSIC (VNCM) algorithm.

Within the *i*th $\left(i=1,\ldots ,\tilde{L}\right)$ frame, vectorizing $R_{rr}\left[i\right]$

(7) $$\begin{array}{c} \begin{array}{c} z^{\prime }\left[i\right]=vec\left(R_{\mathrm{rr}}\left[i\right]\right)=\left(A_{NC}^{*}⊙A_{NC}\right)p\left[i\right]+\sigma_{n}^{2}v^{\prime }, \end{array} \end{array}$$

where $p\left[i\right]={\left[\sigma_{1}^{2}\left[i\right],\sigma_{2}^{2}\left[i\right],\ldots ,\sigma_{Q}^{2}\left[i\right]\right]}^{T}$, $v^{\prime }=vec\left(I\right)$ and the *q*th (q=1,2,…,Q) column vector of $A_{NC}^{*}⊙A_{NC}$ has the form

(8) $$\begin{array}{cc} a_{NC}^{*}\left(\theta_{q}\right)⊗a_{NC}\left(\theta_{q}\right) & ={\left[\begin{array}{c} a\left(\theta_{q}\right)\\ e^{-j\varphi_{q}}a^{*}\left(\theta_{q}\right) \end{array}\right]}^{*}⊗\left[\begin{array}{c} a\left(\theta_{q}\right)\\ e^{-j\varphi_{q}}a^{*}\left(\theta_{q}\right) \end{array}\right]\\ \; & =\left[\begin{array}{c} a^{*}\left(\theta_{q}\right)⊗a\left(\theta_{q}\right)\\ a^{*}\left(\theta_{q}\right)⊗e^{-j\varphi_{q}}a^{*}\left(\theta_{q}\right)\\ e^{j\varphi_{q}}a\left(\theta_{q}\right)⊗a\left(\theta_{q}\right)\\ e^{j\varphi_{q}}a\left(\theta_{q}\right)⊗e^{-j\varphi_{q}}a^{*}\left(\theta_{q}\right) \end{array}\right]=\left[\begin{array}{c} NCdiff1\\ NCsum1\\ NCsum2\\ NCdiff2 \end{array}\right]. \end{array}$$

Having the similar form as the received signal x(t) in (1), $z^{\prime }\left[i\right]$ in (7) can be seen as the equivalent received signal at a virtual array whose steering matrix is given by $A_{NC}^{*}⊙A_{NC}$. According to (8), we can find that the virtual sensor locations can be represented as $\left(d_{r_{1}}-d_{r_{2}}\right)\cup \pm \left(d_{r_{1}}+d_{r_{2}}\right),1\leq r_{1},r_{2}\leq R$. Thus, the novel NC virtual array is a diff–sum coarray consisting of both the difference and sum results. Specially, in (8), NCdiff1 and NCdiff2 correspond to the difference coarray, NCsum1 corresponds to the nonpositive sum coarray and NCsum2 corresponds to the non-negative sum coarray. In the following, we would show that the MNDS of the VNCM method is determined by the DOFs of the diff–sum coarray, regardless of the NC phase $\varphi_{q}$. Assume that the consecutive range of the diff–sum coarray is $\left[-l_{c},l_{c}\right]$. As the performance of the MUSIC-class methods are mainly determined by the ULA part of the virtual array, we remove the repeated and discrete lags in (7) to obtain

(9) $$\begin{array}{c} \begin{array}{c} z\left[i\right]=Bp\left[i\right]+\sigma_{n}^{2}v, \end{array} \end{array}$$

where v is a $(2l_{c}+1)\times 1$ vector extracted from $v^{\prime }$ and $B=[b\left(\theta_{1}\right),b\left(\theta_{2}\right),\ldots ,b\left(\theta_{Q}\right)]$ is a $(2l_{c}+1)\times Q$ matrix with the *q*th column being

(10) $$\begin{array}{c} \begin{array}{c} b\left(\theta_{q}\right)=\left[\begin{array}{c} e^{-j\varphi_{q}}{\tilde{a}}_{1}\left(\theta_{q}\right)\\ {\tilde{a}}_{2}\left(\theta_{q}\right)\\ e^{j\varphi_{q}}{\tilde{a}}_{3}\left(\theta_{q}\right) \end{array}\right]=\tilde{A}\left(\theta_{q}\right)\tilde{\varphi }\left(\theta_{q}\right), \end{array} \end{array}$$

where ${\tilde{a}}_{1}\left(\theta_{q}\right)$, ${\tilde{a}}_{2}\left(\theta_{q}\right)$ and ${\tilde{a}}_{3}\left(\theta_{q}\right)$ are respectively extracted from $a^{*}\left(\theta_{q}\right)⊗a^{*}\left(\theta_{q}\right)$, $a^{*}\left(\theta_{q}\right)⊗a\left(\theta_{q}\right)$ and $a\left(\theta_{q}\right)⊗a\left(\theta_{q}\right)$, $\tilde{A}\left(\theta_{q}\right)=blkdiag\left({\tilde{a}}_{1}\left(\theta_{q}\right),{\tilde{a}}_{2}\left(\theta_{q}\right),{\tilde{a}}_{3}\left(\theta_{q}\right)\right)$ is a block diagonal matrix and $\tilde{\varphi }\left(\theta_{q}\right)={\left[e^{-j\varphi_{q}},1,e^{j\varphi_{q}}\right]}^{T}$.

Combining all the NC virtual received signals $z\left[i\right],i=1,2,\ldots ,\tilde{L}$, we can obtain the NC virtual frame-data matrix over all the $\tilde{L}$ frames as

(11) $$\begin{array}{c} \begin{array}{c} Z=\left[z\left[1\right],z\left[2\right],\ldots ,z\left[\tilde{L}\right]\right]=BP+\sigma_{n}^{2}V, \end{array} \end{array}$$

where $P=\left[p\left[1\right],p\left[2\right],\ldots ,p\left[\tilde{L}\right]\right]$ and $V={\left[v,v,\ldots ,v\right]}_{\left(2l_{c}+1\right)\times \tilde{L}}$. According to A2) in Section 2, it is obvious that each row in P is a wide-sense stationary process with the expectation being ${\bar{m}}_{q}$. Thus, the expectation vector of $z\left[i\right]$ can be expressed as

(12) $$\begin{array}{c} \begin{array}{c} \bar{z}=E\left[z\left[i\right]\right]\approx \frac{1}{\tilde{L}}\sum_{i=1}^{\tilde{L}}z\left[i\right]=B\frac{1}{\tilde{L}}\sum_{i=1}^{\tilde{L}}p\left[i\right]+\sigma_{n}^{2}v=B\bar{p}+\sigma_{n}^{2}v, \end{array} \end{array}$$

where $\bar{p}={\left[{\bar{m}}_{1},{\bar{m}}_{2},\ldots ,{\bar{m}}_{Q}\right]}^{T}$. Subtracting $\bar{z}$ from each column vector of the NC virtual frame-data matrix Z, we have

(13) $$\begin{array}{cc} \tilde{Z} & =Z-\bar{z}{\left[1,1,\ldots ,1\right]}_{1\times \tilde{L}}\\ \; & =B\left(P-\bar{p}{\left[1,1,\ldots ,1\right]}_{1\times \tilde{L}}\right)+\sigma_{n}^{2}V-\sigma_{n}^{2}V\\ \; & =B\tilde{P}, \end{array}$$

where $\tilde{Z}=\left[\tilde{z}\left[1\right],\tilde{z}\left[2\right],\ldots ,\tilde{z}\left[\tilde{L}\right]\right]$ with $\tilde{z}\left[i\right]=z\left[i\right]-\bar{z},i=1,2,\ldots ,\tilde{L}$, and $\tilde{P}=\left[\tilde{p}\left[1\right],\tilde{p}\left[2\right],\ldots ,\tilde{p}\left[\tilde{L}\right]\right]$ with $\tilde{p}\left[i\right]=p\left[i\right]-\bar{p}$. It is obvious that each row in $\tilde{P}$ is a zero-mean wide-sense stationary process. Besides, according to (A2) in Section 2, each row sequence in $\tilde{P}$ is uncorrelated with the other row sequences. Thus, similar to (6), we can obtain the correlation matrix of $\tilde{z}\left[i\right],i=1,2,\ldots ,\tilde{L}$ as

(14) $$\begin{array}{c} \begin{array}{c} R_{\tilde{z}\tilde{z}}=E\left[\tilde{z}\left[i\right]{\tilde{z}}^{H}\left[i\right]\right]\approx \frac{1}{\tilde{L}}\tilde{Z}{\tilde{Z}}^{H}=BR_{\tilde{p}\tilde{p}}B^{H}, \end{array} \end{array}$$

where $R_{\tilde{p}\tilde{p}}=diag\left({\tilde{\sigma }}_{1}^{2},{\tilde{\sigma }}_{2}^{2},\ldots ,{\tilde{\sigma }}_{Q}^{2}\right)$. As $R_{\tilde{p}\tilde{p}}$ is a full-rank matrix, the eigenvalue decomposition of $R_{\tilde{z}\tilde{z}}$ can be obtained as

(15) $$\begin{array}{c} \begin{array}{c} R_{\tilde{z}\tilde{z}}=U_{\tilde{p}}\Sigma_{\tilde{p}}{U_{\tilde{p}}}^{H}+U_{\tilde{n}}\Sigma_{\tilde{n}}{U_{\tilde{n}}}^{H}, \end{array} \end{array}$$

where $U_{\tilde{p}}$ is the signal subspace whose columns represent the signal subspace eigenvectors of $R_{\tilde{z}\tilde{z}}$, $U_{\tilde{n}}$ is the noise subspace whose columns represent the noise subspace eigenvectors of $R_{\tilde{z}\tilde{z}}$, $\Sigma_{\tilde{p}}=diag\left(\lambda_{1},\lambda_{2},\ldots ,\lambda_{Q}\right)$ with $\lambda_{1},\lambda_{2},\ldots ,\lambda_{Q}$ representing the *Q* largest eigenvalues of $R_{\tilde{z}\tilde{z}}$, and $\Sigma_{\tilde{n}}=0_{\left(2l_{c}+1-Q\right)\times \left(2l_{c}+1-Q\right)}$. Due to the orthogonality between the signal subspace and the noise subspace, any direction $\theta_{q},q=1,2,\ldots ,Q$ from $\left\{\theta_{1},\ldots ,\theta_{Q}\right\}$ satisfies the following equation

(16) $$\begin{array}{c} \begin{array}{c} b^{H}\left(\theta_{q}\right)U_{\tilde{n}}U_{\tilde{n}}^{H}b\left(\theta_{q}\right)=0. \end{array} \end{array}$$

Associated with (10), (16) can be rewritten as

(17) $$\begin{array}{c} \begin{array}{c} {\tilde{\varphi }}^{H}\left(\theta_{q}\right){\tilde{A}}^{H}\left(\theta_{q}\right)U_{\tilde{n}}U_{\tilde{n}}^{H}\tilde{A}\left(\theta_{q}\right)\tilde{\varphi }\left(\theta_{q}\right)=0. \end{array} \end{array}$$

According to [1,35,36], ${\tilde{A}}^{H}\left(\theta \right)U_{\tilde{n}}U_{\tilde{n}}^{H}\tilde{A}\left(\theta \right)$ is rank deficient at $\theta =\theta_{q},q=1,2,\ldots ,Q$. Thus, the DOAs can be estimated by the following estimator,

(18) $$\begin{array}{c} \begin{array}{c} f\left(\theta \right)=\frac{1}{det\left\{{\tilde{A}}^{H}\left(\theta \right)U_{\tilde{n}}U_{\tilde{n}}^{H}\tilde{A}\left(\theta \right)\right\}}. \end{array} \end{array}$$

Searching the direction θ over the range −ππ22,ππ22, the DOAs can be obtained from the peaks in $f\left(\theta \right)$.

**Remarks.**  When any element $e^{j2\pi l\sin\left(\theta \right)/\lambda \;}\left(-l_{c}\leq l\leq l_{c}\right)$ in $\tilde{A}\left(\theta \right)$ is replaced by $z^{l}$ ($z=e^{j2\pi \sin\left(\theta \right)/\lambda \;}$), $det\left\{{\tilde{A}}^{H}\left(\theta \right)U_{\tilde{n}}U_{\tilde{n}}^{H}\tilde{A}\left(\theta \right)\right\}$ can be seen as a polynomial of degree $4(l_{c}-1)$, whose roots appear in reciprocal conjugate pairs. Thus, the VNCM algorithm can detect up to $2(l_{c}-1)$ signals. Since the DOF of the diff–sum coarray is $2l_{c}+1$, it can be concluded that MNDS=DOF−3 for the VNCM method. From the discussions above, it is obvious that the MNDS of the VNCM method is determined by the DOF of the diff–sum coarray, regardless of the NC phase. With R physical sensors, the MNDS of the traditional NC DOA estimators is 2(R−1). As $l_{c}$ is obtained by combining the difference and sum results of the physical sensor locations, we can conclude $l_{c}>R$. Therefore, the VNCM algorithm can detect more signals than the traditional NC DOA estimators. Furthermore, designing a sparse array, of which the diff–sum coarray achieves a high number of DOFs, can help further improve the performance of the proposed method. It is noted that in theory, the high-order cumulant-based MUSIC method can also be applied to the sparse array to detect NC sources. The virtual array generated in this kind of method should be able to achieve the same number of DOFs as that generated in the VNCM method. However, this kind of method should first solve one issue, which is how to separate the NC phases from the cumulant matrix to perform the eigenvalue decomposition. The method proposed in this paper can help to solve this issue, which would be a future work for us to do. The difference between the NC high-order cumulant-based method and the VNCM method is that the NC high-order cumulant-based method is restricted to non-Gaussian signal sources, but the signal model in the VNCM method is not necessarily non-Gaussian.

## 4. The Diff–Sum Coprime Array with Multiperiod Subarrays Based on the Concept of the Diff–Sum Coarray

Now, since the difference set and the sum set for the coprime array are complementary [16,37], we choose the CAMpS [30,31] as the basic array model. In this section, we would summarize and prove the properties and the DOF of the diff–sum coarray for the CAMpS. Then, based on these properties, we improve the CAMpS to propose a diff–sum coprime array with multiperiod subarrays (DsCAMpS) of which the diff–sum coarray achieves a higher number of DOFs.

### 4.1. The CAMpS and the Concept of the Diff–Sum Coarray

**Figure 1:**
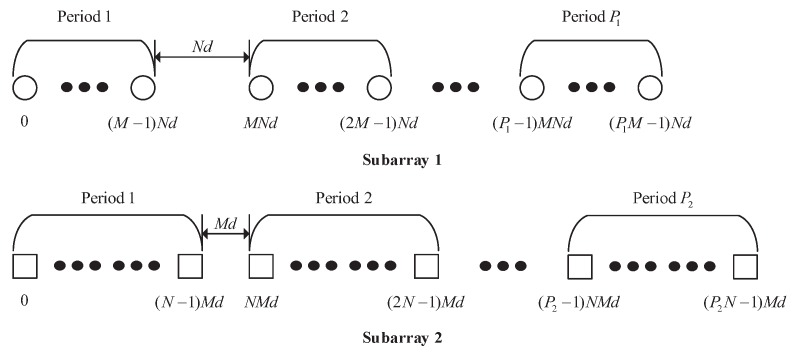
The coprime array with multiperiod subarrays.

Firstly, we have a quick review of the CAMpS. As shown in Figure 1, the CAMpS, which is the multiperiod extension of the prototype coprime array, consists of two uniform linear subarrays. Subarray 1 contains $P_{1}M$ sensors with the intersensor spacing of *N* units, and Subarray 2 contains $P_{2}N$ sensors with the intersensor spacing of *M* units. We use *d* to denote the unit interelement spacing. Then, the sensors of the CAMpS are located at $S_{CAMpS}=S_{1}\cup S_{2}$, where $S_{1}=\left\{mNd\left|0\leq m\leq P_{1}M-1\right.\right\}$ and $S_{2}=\left\{nMd\left|0\leq n\leq P_{2}N-1\right.\right\}$. Due to *M* and *N* being coprime, there are $\min\{P_{1},P_{2}\}$ common elements between the two subarrays. Hence, the number of the elements in the CAMpS is $P_{1}M+P_{2}N-\min\left\{P_{1},P_{2}\right\}$. For convenience, in the following sections of this paper, we normalize all the locations by the unit interelement spacing *d*.

Applying the VNCM method to the CAMpS, the resulting virtual array can be represented as

(19) $$\begin{array}{c} \begin{array}{c} S_{ds}=S_{diff}\cup S_{sum}^{+}\cup S_{sum}^{-}\;, \end{array} \end{array}$$

where $S_{ds}$ is the diff–sum set, $S_{diff}=\left\{d_{r_{1}}-d_{r_{2}}\left|1\leq r_{1},r_{2}\leq R\right.\right\}$ is the difference set, $S_{sum}^{+}=\left\{d_{r_{1}}+d_{r_{2}}\left|1\leq r_{1},r_{2}\leq R\right.\right\}$ is the non-negative sum set and $S_{sum}^{-}=\left\{-\left(d_{r_{1}}+d_{r_{2}}\right)\left|1\leq r_{1},r_{2}\leq R\right.\right\}$ is the nonpositive sum set.

When choosing the CAMpS in Figure 1 as the basic array model, $S_{diff}$ can be expressed as the following union set,

(20) $$\begin{array}{c} \begin{array}{c} S_{diff}=S_{cd}\cup S_{cd}^{-}\cup S_{sd}\cup S_{sd}^{-}\;, \end{array} \end{array}$$

where $S_{cd}=\left\{d_{S_{2}}-d_{S_{1}}\left|d_{S_{2}}\in S_{2},d_{S_{1}}\in S_{1}\right.\right\}$ is the cross-difference set between $S_{2}$ and $S_{1}$, $S_{cd}^{-}=\left\{d_{S_{1}}-d_{S_{2}}\right\}$ is the mirrored set of $S_{cd}$, $S_{sd}=\left\{d_{S_{1}}\right\}\cup \left\{d_{S_{2}}\right\}$ is the self-difference set of $S_{1}$ and $S_{2}$ and $S_{sd}^{-}=\left\{-d_{S_{1}}\right\}\cup \left\{-d_{S_{2}}\right\}$ is the mirrored set of $S_{sd}$. Similarly, the total sum set $S_{sum}=S_{sum}^{+}\cup S_{sum}^{-}$ can also be expressed as a union set

(21) $$\begin{array}{c} \begin{array}{c} S_{sum}=\underset{S_{sum}^{+}}{\underset{︸}{S_{cs}^{+}\cup S_{ss}^{+}}}\cup \underset{S_{sum}^{-}}{\underset{︸}{S_{cs}^{-}\cup S_{ss}^{-}}}\;, \end{array} \end{array}$$

where $S_{ss}^{+}=\left\{d_{S_{1}}+{d^{\prime }}_{S_{1}}\left|d_{S_{1}},{d^{\prime }}_{S_{1}}\in S_{1}\right.\right\}\cup \left\{d_{S_{2}}+{d^{\prime }}_{S_{2}}\left|d_{S_{2}},{d^{\prime }}_{S_{2}}\in S_{2}\right.\right\}$ is the self-sum set of $S_{1}$ and $S_{2}$, $S_{ss}^{-}=\left\{-\left(d_{S_{1}}+{d^{\prime }}_{S_{1}}\right)\right\}\cup \left\{-\left(d_{S_{2}}+{d^{\prime }}_{S_{2}}\right)\right\}$ is the mirrored set of $S_{ss}^{+}$, $S_{cs}^{+}=\left\{d_{S_{1}}+d_{S_{2}}\right\}$ is the cross-sum set between $S_{1}$ and $S_{2}$, $S_{cs}^{-}=\left\{-\left(d_{S_{1}}+d_{S_{2}}\right)\right\}$ is the mirrored set of $S_{cs}^{+}$.

### 4.2. The Properties of the Diff–Sum Set for the CAMpS

Without loss of generality, we assume the period $P_{1}$ of Subarray 1 is no larger than the period $P_{2}$ of Subarray 2, that is, $P_{2}\geq P_{1}\geq 1$. Denote $S_{1i}=\left\{Nm\left|\left(i-1\right)M\leq m\leq iM-1\right.\right\}$ and $S_{2j}=\left\{Mn\left|\left(j-1\right)N\leq n\leq jN-1\right.\right\}$ as the location set of Period *i*$\left(1\leq i\leq P_{1}\right)$ in Subarray 1 and the location set of Period *j*$\left(1\leq j\leq P_{2}\right)$ in Subarray 2.

According to [30,31], some properties of the difference set for the CAMpS have been summarised as follows:

(1) $S_{cd}$ contains all the consecutive lags in the range $\left[-\left(P_{1}-1\right)MN-M+1,\left(P_{2}-1\right)MN+N-1\right]$.
(2) When $P_{2}>P_{1}$, the difference set $S_{diff}$ contains all the consecutive lags in the range $\left[-\left(P_{2}-1\right)MN-N+1,\left(P_{2}-1\right)MN+N-1\right]$.
(3) When $P_{2}=P_{1}$, the result becomes $\left[-\left(P_{2}-1\right)MN-M-N+1,\left(P_{2}-1\right)MN+M+N-1\right]$.

Combining these properties, we can find that when *M* and *N* are fixed, the maximum value $s_{cdmax}$ in the consecutive range of $S_{cd}$ is determined by the period $P_{2}$ of Subarray 2, and the minimum value is determined by the period $P_{1}$ of Subarray 1. Considering the consecutive range of $S_{diff}$, when $P_{2}>P_{1}$, both the minimum and the maximum have the same absolute value as $s_{cdmax}$ and the two values are only related to $P_{2}$. When $P_{2}=P_{1}$, the two values have greater absolute value than $s_{cdmax}$ since some holes in $S_{diff}$ with $P_{1}=P_{2}-1$ can be filled by the cross-difference results between $S_{1P_{2}}$ and $S_{2}$. Thus, we can obtain the following two conclusions: (1) When $P_{2}>P_{1}\geq 1$, the difference set for the CAMpS has the same consecutive range as that for a simplified CAMpS of which the two subarrays are respectively $S_{11}$ and $S_{2}$. (2) When $P_{2}=P_{1}\geq 1$, the equivalent array is a simplified CAMpS of which the two subarrays are respectively $S_{11}\cup S_{1P_{1}}$ and $S_{2}$.

**Figure 2:**
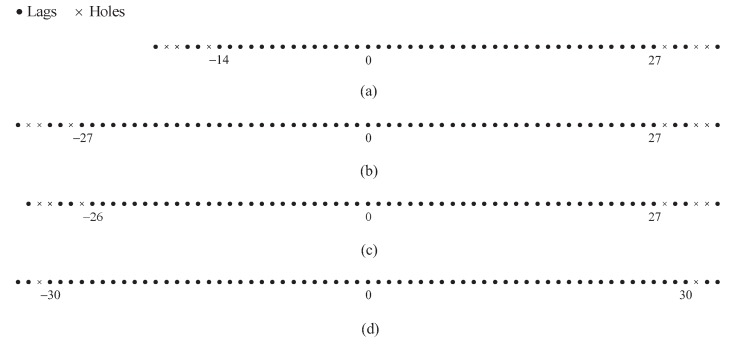
Two examples of the difference coarray for the CAMpS with (M,N)=(3,4): (**a**) the set $S_{cd}$ with $\left(P_{1},P_{2}\right)=\left(2,3\right)$; (**b**) the set $S_{diff}$ with $\left(P_{1},P_{2}\right)=\left(2,3\right)$; (**c**) the set $S_{cd}$ with $\left(P_{1},P_{2}\right)=\left(3,3\right)$; and (**d**) the set $S_{diff}$ with $\left(P_{1},P_{2}\right)=\left(3,3\right)$.

In Figure 2, we assume (M,N)=(3,4) and show two examples of the difference sets for the CAMpS with $\left(P_{1},P_{2}\right)=\left(2,3\right)$ and $\left(P_{1},P_{2}\right)=\left(3,3\right)$ as the illustrative examples of the above properties. Figure 2 shows that the difference coarray is symmetrical with the zero point as the center. When $P_{2}>P_{1}$, the number of the consecutive elements in the difference coarray is $2s_{cdmax}+1$. When $P_{1}$ increases to $P_{2}$, the result becomes $2s_{cdmax}+2M+1$.

The following proposition reveals the properties of the non-negative sum set for the CAMpS. The properties of the nonpositive sum set can be deduced by reversing the results of the following proposition.

**Proposition** **1.** *The following facts hold for $S_{cs}^{+}$ and $S_{sum}^{+}$:*

*(a)*  The consecutive range of $S_{cs}^{+}$ is $\left[\left(M-1\right)\left(N-1\right),\left(P_{1}+P_{2}-1\right)MN-1\right]$.
*(b)*  When $P_{2}>P_{1}$, the non-negative sum set $S_{sum}^{+}$ contains all the consecutive lags in the range $\left[\left(M-1\right)\left(N-1\right),\left(P_{1}+P_{2}-1\right)MN+N-1\right]$.
*(c)*  When $P_{2}=P_{1}$: $S_{sum}^{+}$ with M=2 contains all the consecutive lags in the range $\left[\left(M-1\right)\left(N-1\right),\left(P_{1}+P_{2}-1\right)MN+N-1\right]$; when N=2, the result becomes $\left[\left(M-1\right)\left(N-1\right),\left(P_{1}+P_{2}-1\right)MN+M-1\right]$; when M,N>2, the result is $\left[\left(M-1\right)\left(N-1\right),\left(P_{1}+P_{2}-1\right)MN+M+N-1\right]$.
*(d)*  The non-negative sum set for the CAMpS has the same consecutive range as that for a simplified CAMpS whose two subarrays are respectively $S_{11}\cup S_{1P_{1}}$ and $S_{2}$.

**Proof.**  See Appendix A. ☐

**Figure 3:**
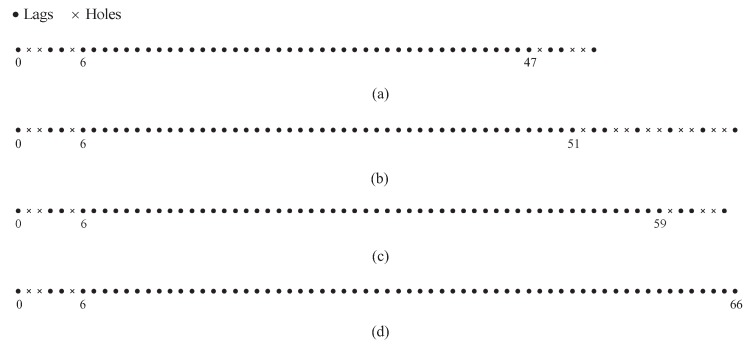
Two examples of the non-negative sum coarray for the CAMpS with (M,N)=(3,4): (**a**) the set $S_{cs}^{+}$ with $\left(P_{1},P_{2}\right)=\left(2,3\right)$; (**b**) the set $S_{sum}^{+}$ with $\left(P_{1},P_{2}\right)=\left(2,3\right)$; (**c**) the set $S_{cs}^{+}$ with $\left(P_{1},P_{2}\right)=\left(3,3\right)$; and (**d**) the set $S_{sum}^{+}$ with $\left(P_{1},P_{2}\right)=\left(3,3\right)$.

In the proof of Proposition 1, we have proved that some holes in the cross-sum set can be aligned with the elements in the self-sum set. Thus, $S_{sum}^{+}$ has a wider consecutive range than $S_{cs}^{+}$. In addition, when *M* and *N* are fixed, the maximums in the consecutive range of $S_{cs}^{+}$ and $S_{sum}^{+}$ are determined by the periods of the two subarrays and the minimum is fixed. In Figure 3, we assume (M,N)=(3,4) and show two examples of the non-negative sum coarray for the CAMpS with $\left(P_{1},P_{2}\right)=\left(2,3\right)$ and $\left(P_{1},P_{2}\right)=\left(3,3\right)$ as the illustrative examples of the above properties. It is obvious that when the period of one subarray adds 1, the maximums in the consecutive range of $S_{cs}^{+}$ and $S_{sum}^{+}$ would add O(MN). Since $S_{sum}^{-}$ is the mirrored set of $S_{sum}^{+}$, the consecutive range of the total sum set $S_{sum}=S_{sum}^{+}\cup S_{sum}^{-}$ is symmetrical with the zero point as the center. Then, combining Proposition 1 and the properties of the difference set, we give the properties of the diff–sum set $S_{ds}$ for the CAMpS in the following proposition. Defining $s_{ds\max1}=\left(P_{1}+P_{2}-1\right)MN+N-1$, $s_{ds\max2}=\left(P_{1}+P_{2}-1\right)MN+M-1$ and $s_{ds\max3}=\left(P_{1}+P_{2}-1\right)MN+M+N-1$, we conclude:

**Proposition** **2.** When $P_{2}>P_{1}$, the consecutive range of $S_{ds}$ for the CAMpS is $\left[-s_{ds\max1},s_{ds\max1}\right]$. When $P_{2}=P_{1}$, the consecutive range of $S_{ds}$ is summarised based on the following three cases: (1) when M=2, the consecutive range is $\left[-s_{ds\max1},s_{ds\max1}\right]$; (2) when N=2, the consecutive range is $\left[-s_{ds\max2},s_{ds\max2}\right]$; (3) when M,N>2, the consecutive range is $\left[-s_{ds\max3},s_{ds\max3}\right]$.

According to Proposition 2, in Table 1, we summarize the DOF (the number of the consecutive lags) of $S_{ds}$. The corresponding MNDS of the VNCM method can also be obtained by the equation MNDS = DOF−3.

### 4.3. The Diff–Sum Coprime Array with Multiperiod Subarrays

**Figure 4:**
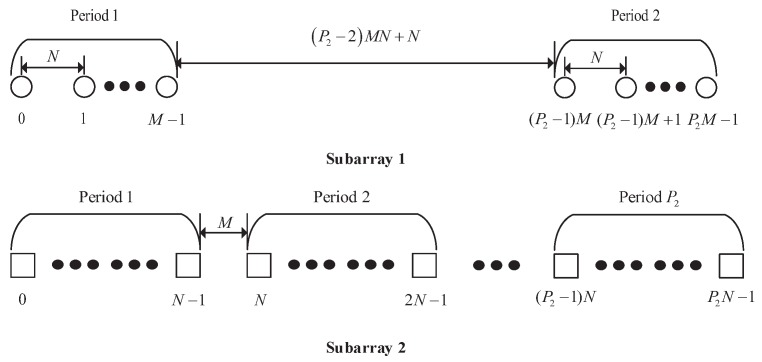
The diff–sum coprime array with multiperiod subarrays.

According to the properties of the difference set, Propositions 1 and 2, the consecutive range of $S_{ds}$ for the CAMpS is the same as that of $S_{ds}$ for a simplified CAMpS, of which the location sets of the two subarrays can be expressed as $S_{11}\cup S_{1P_{1}}$ and $S_{2}$. We name the equivalent array as the diff–sum coprime array with multiperiod subarrays (DsCAMpS). Since the DOF of the DsCAMpS with $P_{1}=P_{2}$ is greater than that with $P_{1}<P_{2}$, we define $P_{1}=P_{2}$ in the DsCAMpS. Then, the structure of the DsCAMpS can be shown in Figure 4.

When $P_{2}\geq 2$, Subarray 1 in the DsCAMpS contains 2 periods, of which each period contains *M* sensors with the intersensor spacing of *N* units. The displacement between the two periods is $(P_{2}-2)MN+N$. Subarray 2 in the DsCAMpS contains $P_{2}N$ sensors with the intersensor spacing of *M* units. The location set of the DsCAMpS can be expressed as

(22) $$\begin{array}{cc} S_{DsCAMpS}= & \left\{mN\left|0\leq m\leq M-1,\left(P_{2}-1\right)M\leq m\leq P_{2}M-1\right.\right\}\\ \; & \cup \left\{nM\left|0\leq n\leq P_{2}N-1\right.\right\}. \end{array}$$

Between the two subarrays, there are two common elements which locate at 0 and $(P_{2}-1)MN$. Thus, the number of the elements in the DsCAMpS is $2M+P_{2}N-2$. When $P_{2}=1$, the DsCAMpS becomes the prototype coprime array with M+N−1 sensors, which means the prototype coprime array is a special kind of the DsCAMpS. Defining $s_{ds\max a}=\left(2P_{2}-1\right)MN+N-1$, $s_{ds\max b}=\left(2P_{2}-1\right)MN+M-1$ and $s_{ds\max c}=\left(2P_{2}-1\right)MN+M+N-1$, we can show the DOF of the diff–sum coarray for the DsCAMpS in Table 2. Compared with the CAMpS, the DsCAMpS can achieve a higher number of DOFs when $P_{2}>2$. When $P_{1}=P_{2}\leq 2$, the two arrays have the same structure and DOF.

**Figure 5:**
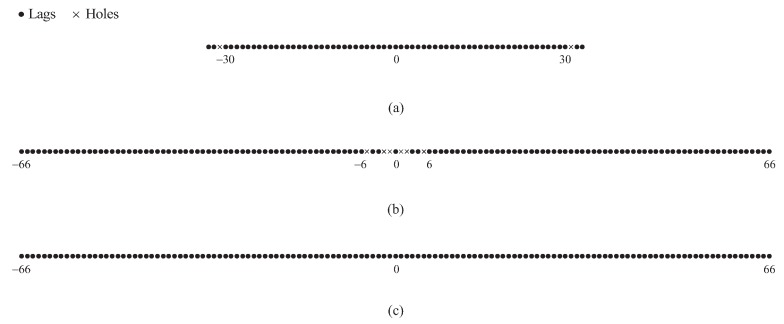
The consisting sets of the diff–sum coarray for the DsCAMpS with $\left(M,N,P_{2}\right)=\left(4,3,3\right)$: (**a**) the difference set $S_{diff}$; (**b**) the total sum set $S_{sum}$; and (**c**) the diff–sum set $S_{ds}$.

In Figure 5, we depict the consisting sets of the diff–sum coarray for the DsCAMpS with $\left(M,N,P_{2}\right)=\left(4,3,3\right)$. Figure 5a shows that the consecutive range of the difference set is [−30,30], which is symmetrical with the zero point as the center. Figure 5b depicts the total sum set $S_{sum}=S_{sum}^{+}\cup S_{sum}^{-}$, which contains all the consecutive lags in the range $\left[-66,-6\right]\cup \left[6,66\right]$. The consecutive range of the difference set for the DsCAMpS with $P_{2}>2$ overlaps with part of the consecutive range of the total sum set. Thus, as shown in Figure 5c, the diff–sum set, which is the union set of $S_{diff}$ and $S_{sum}$, contains all the consecutive lags in the range [−66,66]. Figure 5 verifies the properties of the diff–sum coarray for the DsCAMpS and the complementarity between the corresponding difference set and sum set.

## 5. Simulation Results

In this section, we consider the sensor number of all configurations as R=15. The unit interelement spacing is d=λ/2. Since the DsCAMpS and the CAMpS are the same when $P_{1}=P_{2}\leq 2$, we consider the period $P_{2}$ in Subarray 2 of both the CAMpS and the DsCAMpS satisfying $P_{2}>2$. The configurations utilized in this section are respectively the CAMpS with $\left(M,N,P_{1},P_{2}\right)=\left(4,3,2,3\right)$, the DsCAMpS with $\left(M,N,P_{2}\right)=\left(4,3,3\right)$ and the ULA ={0,1…,14}.

### 5.1. DOF Comparison

**Figure 6:**
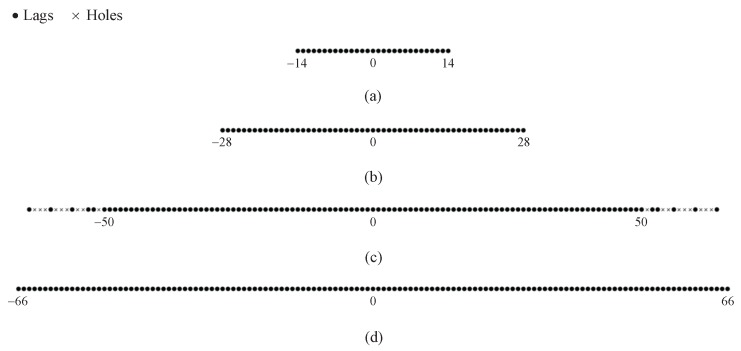
Four virtual configurations (R=15): (**a**) the traditional NC virtual array for the ULA; (**b**) the diff–sum coarray for the ULA; (**c**) the diff–sum coarray for the CAMpS with $\left(M,N,P_{1},P_{2}\right)=\left(4,3,2,3\right)$; and (**d**) the diff–sum coarray for the DsCAMpS with $\left(M,N,P_{2}\right)=\left(4,3,3\right)$.

Figure 6 depicts four virtual configurations, which are respectively the traditional NC virtual array for the ULA, the diff–sum coarrays for the ULA, the CAMpS and the DsCAMpS. The traditional NC virtual array is constructed in the NC MUSIC algorithm and the three diff–sum coarrays are obtained by using the VNCM algorithm. As shown in Figure 6a, the traditional NC virtual array for the ULA, which consists of the physical array and its flipped array, contains all the consecutive lags in the range [−14,14]. In Figure 6b–d, the consecutive ranges of the three diff–sum coarrays for the ULA, the CAMpS and the DsCAMpS are respectively [−28,28], [−50,50] and [−66,66]. Comparing Figure 6a,b, it is obvious that the diff–sum coarray achieves a higher number of DOFs than the traditional NC virtual array. From Figure 6b–d, we can find that the diff–sum coarray constructed by using the sparse array has a larger consecutive range than that constructed by using the ULA. In addition, the diff–sum coarray for the DsCAMpS contains more consecutive lags than that for the CAMpS with the same number of sensors.

### 5.2. MUSIC Spectra

**Figure 7:**
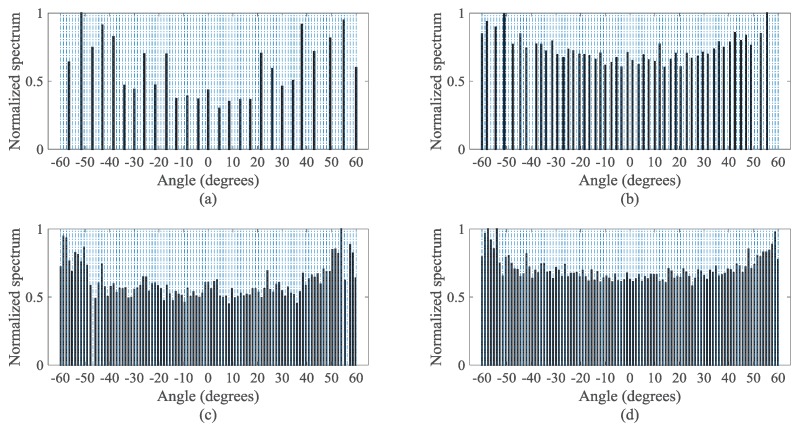
Spatial spectra of the uniformly distributed signals detected by the four virtual configurations (Q=101 and SNR = 10 dB): (**a**) the ULA with the NC MUSIC used; (**b**) the ULA with the VNCM used; (**c**) the CAMpS with the VNCM used; and (**d**) the DsCAMpS with the VNCM used.

Figure 7 presents the normalized MUSIC spectra of the uniform distributed signals detected by the four virtual configurations in Figure 6. Here, the frame length *L*, the number $\tilde{L}$ of the frames in the VNCM method and the snapshots $L_{s}$ in the NC MUSIC satisfy $L=\tilde{L}=L_{s}=800$. We consider the input SNR = 10 dB, and we suppose Q=101 sources, which are uniformly distributed between $-60^{\circ }$ and $60^{\circ }$. It is noted that the frame length is actually the snapshots in one frame.

Figure 7d shows that the DsCAMpS with the VNCM used can detect all the 101 sources since the DOF of the diff–sum coarray for the DsCAMpS is 133. The corresponding MNDS is 130. According to Figure 7a–c, the other three virtual configurations fail to obtain the correct DOA estimations. This is because the numbers of the consecutive lags in the traditional NC virtual array for the ULA, the diff–sum coarrays for the ULA and the CAMpS are respectively 29, 57 and 101. Then, the corresponding MNDSs are respectively 28, 54 and 98. Thus, with the same number of sensors, the DsCAMpS with the VNCM used achieves a better performance than the ULA with the NC MUSIC used, the ULA with the VNCM used and the CAMpS with the VNCM used.

**Figure 8:**
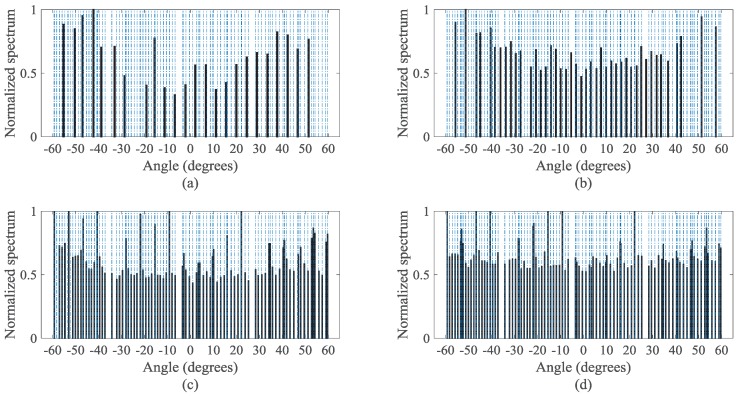
Spatial spectra of the non-uniformly distributed signals detected by the four virtual configurations (Q=101 and SNR = 10 dB): (**a**) the ULA with the NC MUSIC used; (**b**) the ULA with the VNCM used; (**c**) the CAMpS with the VNCM used; and (**d**) the DsCAMpS with the VNCM used.

In order to demonstrate the estimated DOAs when the signal source distribution changes, in Figure 8, we further simulate the normalized MUSIC spectra of the non-uniformly distributed signals detected by the four virtual configurations. Here, Q=101 sources are non-uniformly distributed between $-60^{\circ }$ and $60^{\circ }$, and the other parameters are the same as those in Figure 7. The non-uniform distribution of sources could make some DOAs get very close, which may deteriorate the DOA estimation performance. However, Figure 8d shows that the DsCAMpS with the VNCM used can still detect all the sources. In contrast, the other three virtual configurations fail to obtain the correct DOA estimations, which is shown in Figure 8a–c. Thus, regardless of whether the signal sources are uniformly distributed or non-uniformly distributed, the DsCAMpS with the VNCM used achieves a better performance than the other three virtual arrays.

### 5.3. Root Mean Square Error (RMSE)

**Figure 9:**
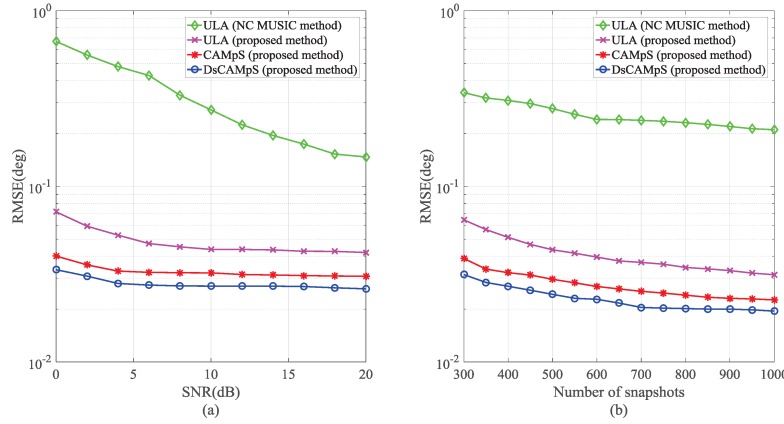
Estimation precision (Q=25): (**a**) RMSE versus SNR; and (**b**) RMSE versus the number of snapshots.

We further conduct Monte Carlo simulations to compare the DOA estimation performance of the four virtual configurations in Figure 6. Here, we use the RMSE of the estimated DOAs as the performance metric. The RMSE is defined as

(23) $$\begin{array}{c} \begin{array}{c} RMSE=\sqrt{\frac{1}{JQ}\sum_{j=1}^{J}\sum_{q=1}^{Q}{\left({\hat{\theta }}_{q}\left(j\right)-\theta_{q}\right)}^{2}}, \end{array} \end{array}$$

where *J* is the number of Monte Carlo simulations, $\theta_{q}$ denotes the real DOA of the *q*th signal source and ${\hat{\theta }}_{q}\left(j\right)$ denotes the estimate of $\theta_{q}$ for the *j*th trial, j=1,…,J. In all the simulations, we consider the signal source number Q=25 and utilize 500 independent Monte Carlo simulations. Figure 9a depicts the RMSE performance as a function of the input SNR. In this simulation, we suppose $L=\tilde{L}=L_{s}=500$. It is clear that all the arrays with the VNCM used outperform the ULA with the NC MUSIC used due to the diff–sum coarray containing more consecutive elements than the traditional NC virtual array. Among the three arrays with the VNCM used, the DsCAMpS achieves the best performance. In Figure 9b, we suppose SNR = 10 dB to compare the RMSE performance as a function of the snapshots. Here, we consider $L=\tilde{L}=L_{s}$. From the results, all the arrays with the VNCM used still perform much better than the ULA with the NC MUSIC used. Compared with the RMSE of the ULA and the CAMpS with the VNCM used, the RMSE of the DsCAMpS with the VNCM used is smaller. The results of the two simulations suggest that the diff–sum coarray achieves a higher number of DOF than the traditional NC virtual array and the DsCAMpS is a novel array of which the diff–sum coarray has higher DOF than that of the ULA and the CAMpS.

## 6. Conclusions

We have proposed the VNCM method, which utilizes both the NC property and the concept of the coarray, to obtain a novel NC virtual array named as the diff–sum coarray. Due to comprising both the difference set and the sum set, the diff–sum coarray has a higher DOF than the traditional NC virtual array. Also, we utilize the quasi-stationary characteristic instead of the spatial smoothing method to solve the coherent issue generated by the KR product operation. Thus, the available DOFs would not be reduced by half. Taking the CAMpS as the array model, we have summarized the properties of the corresponding diff–sum coarray and then further proposed the DsCAMpS to achieve a higher number of DOFs. The high DOF of the diff–sum coarray and the performance of the novel method were numerically studied and evaluated.

## Figures and Tables

**Table 1:** The DOFs of the diff–sum coarray for the CAMpS.

	$P_{2}>P_{1}\geq 1$	$P_{2}=P_{1}\geq 1$		
	M,N≥2	N>M=2	M>N=2	M,N>2
DOF	$2s_{ds\max1}+1$	$2s_{ds\max1}+1$	$2s_{ds\max2}+1$	$2s_{ds\max3}+1$

**Table 2:** The DOFs of the diff–sum coarray for the DsCAMpS .

	N>M=2	M>N=2	M,N>2
DOF	$2s_{ds\max a}+1$	$2s_{ds\max b}+1$	$2s_{ds\max c}+1$

## Appendix A. Proof of Proposition 1

(a) Define $S_{csl}^{+}=\left\{Mn+Nm\left|0\leq n\leq P_{2}N-1,0\leq m\leq M-1\right.\right\}$ as the cross-sum set between $S_{2}$ and $S_{11}$. Firstly, we would show that $S_{csl}^{+}$ contains all the consecutive lags in the range $\left[\left(M-1\right)\left(N-1\right),P_{2}MN-1\right]$. Given an arbitrary integer *x* in the above range, we need to demonstrate there exist $m_{2}\in \left[0,M-1\right]$ and $n_{2}\in \left[0,P_{2}N-1\right]$ such that $x=Mn_{2}+Nm_{2}$. According to [22], we know that there exist $n^{\prime }$ and $m^{\prime }$, which can vary over all the integers, such that $x=Mn^{\prime }+Nm^{\prime }$. Then, we can further have $x=M\left(n^{\prime }+yN\right)+N\left(m^{\prime }-yM\right)$, where *y* is an arbitrary integer. Denoting $n_{2}=n^{\prime }+yN$ and $m_{2}=m^{\prime }-yM$, we can choose the value of *y* to make $m_{2}$ satisfy $0\leq m_{2}\leq M-1$. Thus, we obtain $1-M\leq Mn_{2}=x-Nm_{2}\leq P_{2}MN-1$, that is, $0\leq n_{2}\leq P_{2}N-1$. This demonstrates $S_{csl}^{+}$ contains all the consecutive lags in the range $\left[\left(M-1\right)\left(N-1\right),P_{2}MN-1\right]$. Then, it is obvious that the consecutive range of the cross-sum set between $S_{2}$ and $S_{1i}$ is $\left[\left(M-1\right)\left(N-1\right)+\left(i-1\right)MN,P_{2}MN-1+\left(i-1\right)MN\right]$. When $P_{2}\geq 2$, the length of the consecutive range is greater than MN. Thus, the cross-sum set between $S_{2}$ and $S_{1}$ contains all the consecutive lags in the range $\left[\left(M-1\right)\left(N-1\right),\left(P_{1}+P_{2}-1\right)MN-1\right]$.

(b) In [16], we have proved that in the range $\left[MN,MN+M+N-1\right]$, the holes in the cross-sum result $\left\{Mn+Nm\left|0\leq n\leq N-1,0\leq m\leq M-1\right.\right\}$ between the two subarrays of the coprime array can be expressed as $S_{ph1}\cup S_{ph2}$, where

(A1) $$\begin{array}{c} \begin{array}{c} S_{ph1}=\left\{Mn\left|N\leq n\leq \left(MN+M+N-1\right)/M\;\right.\right\} \end{array} \end{array}$$

and

(A2) $$\begin{array}{c} \begin{array}{c} S_{ph2}=\left\{Nm\left|M\leq m\leq \left(MN+M+N-1\right)/N\;\right.\right\}. \end{array} \end{array}$$

In the CAMpS, Period 1 of Subarray 1 is actually the first subarray of the coprime array and Period 1 of Subarray 2 is the second subarray of the coprime array. In addition, the elements in $S_{1P_{1}}$ are equivalent to the elements in $S_{11}$ adding $(P_{1}-1)MN$, and the elements in $S_{2P_{2}}$ are equivalent to the elements in $S_{21}$ adding $(P_{2}-1)MN$. Thus, in the range $\left[\left(P_{1}+P_{2}-1\right)MN,\left(P_{1}+P_{2}-1\right)MN+M+N-1\right]$, the holes in the cross-sum set between $S_{1P_{1}}$ and $S_{2P_{2}}$ can be expressed as $S_{mh1}\cup S_{mh2}$, where

(A3) $$\begin{array}{c} \begin{array}{c} S_{mh1}=\left\{Mn\left|\left(P_{1}+P_{2}-1\right)N\leq n\leq \left(\left(P_{1}+P_{2}-1\right)MN+M+N-1\right)/M\;\right.\right\} \end{array} \end{array}$$

and

(A4) $$\begin{array}{c} \begin{array}{c} S_{mh2}=\left\{Nm\left|\left(P_{1}+P_{2}-1\right)M\leq m\leq \left(\left(P_{1}+P_{2}-1\right)MN+M+N-1\right)/N\;\right.\right\}. \end{array} \end{array}$$

When $P_{2}>P_{1}$, the self-sum set of Subarray 2 contains all the elements in $S_{mh1}$. However, any element in $S_{mh2}$ is larger than the maximum in the self-sum set of Subarray 1. Therefore, only the holes in the range $\left[\left(P_{1}+P_{2}-1\right)MN,\left(P_{1}+P_{2}-1\right)MN+N-1\right]$ can be filled by the self-sum set of the CAMpS. Then, the consecutive range of the non-negative sum set $S_{sum}^{+}$ is $\left[\left(M-1\right)\left(N-1\right),\left(P_{1}+P_{2}-1\right)MN+N-1\right]$ when $P_{2}>P_{1}$.

(c) Denote

(A5) $$\begin{array}{c} \begin{array}{c} S_{psum}^{+}=\left\{Mn+Nm\right\}\cup \left\{Mn+Mn\right\}\cup \left\{Nm+Nm\right\},0\leq n\leq N-1,0\leq m\leq M-1 \end{array} \end{array}$$

as the sum result for the prototype coprime array. In [16], we have proved that when M=2 or N=2, the consecutive range of $S_{psum}^{+}$ is $\left[\left(M-1\right)\left(N-1\right),MN+N-1\right]$ or $\left[\left(M-1\right)\left(N-1\right),MN+M-1\right]$. Thus, when $P_{2}=P_{1}$, we can know that with M=2, the cross-sum result between $S_{1P_{1}}$ and $S_{2P_{2}}$ contains all the consecutive lags in the range $\left[\left(P_{1}+P_{2}-1\right)MN-M-N+1,\left(P_{1}+P_{2}-1\right)MN+N-1\right]$. According to the property (a) of Proposition 1, it is obvious that the consecutive range of $S_{sum}^{+}$ is $\left[\left(M-1\right)\left(N-1\right),\left(P_{1}+P_{2}-1\right)MN+N-1\right]$. When N=2, the result becomes $\left[\left(M-1\right)\left(N-1\right),\left(P_{1}+P_{2}-1\right)MN+M-1\right]$. When M,N≠2, the set $S_{psum}^{+}$ contains all the consecutive lags in the range $\left[\left(M-1\right)\left(N-1\right),MN+M+N-1\right]$. Then, we can utilize the similar method as the above procedure to demonstrate the consecutive range of $S_{sum}^{+}$ is $\left[\left(M-1\right)\left(N-1\right),\left(P_{1}+P_{2}-1\right)MN+M+N-1\right]$.

(d) In the proof of the property (a), we have shown that the consecutive range of the cross-sum set between $S_{2}$ and $S_{1i}$ is $\left[\left(M-1\right)\left(N-1\right)+\left(i-1\right)MN,P_{2}MN-1+\left(i-1\right)MN\right]$. It is easy to find that the minimum in the consecutive range of the cross-sum set between $S_{2}$ and $S_{1P_{1}}$ is less than the maximum in the consecutive range of the cross-sum set between $S_{2}$ and $S_{11}$. Thus, the consecutive range of the non-negative sum set for the CAMpS is the same as that of the non-negative sum set for a simplified CAMpS, of which the location sets of the two subarrays can be expressed as $S_{11}\cup S_{1P_{1}}$ and $S_{2}$.

## References

[1] Chargé P., Wang Y., Saillard J. (2001). A non-circular sources direction finding method using polynomial rooting. Signal Process..

[2] Shen L., Liu Z., Gou X., Xu Y. (2014). Polynomial-rooting based fourth-order MUSIC for direction-of-arrival estimation of noncircular signals. Syst. Eng. Electron..

[3] Wang Z., Xiaofei Z., Huapu S., Renzheng C. (2017). Non-circular generalised-ESPRIT algorithm for direction of arrival estimation. IET Radar Sonar Navig..

[4] Steinwandt J., Roemer F., Haardt M. Analytical performance assessment of esprit-type algorithms for coexisting circular and strictly non-circular signals. Proceedings of the 2016 IEEE International Conference on Acoustics, Speech and Signal Processing (ICASSP).

[5] Yin J., Wang D., Wu Y., Yao X. (2017). ML-based single-step estimation of the locations of strictly noncircular sources. Digit. Signal Process..

[6] Shi H., Leng W., Guan Z., Jin T. (2017). Two novel two-stage direction of arrival estimation algorithms for two-dimensional mixed noncircular and circular sources. Sensors.

[7] Wang Q., Zhu X., Chen H., Wang L., Yan W., Fang H. (2017). Computationally efficient direction finding for a mixture of circular and strictly noncircular sources with uniform rectangular arrays. Sensors.

[8] Wang X., Wang W., Li X., Liu Q., Liu J. (2016). Sparsity-aware DOA estimation scheme for noncircular source in MIMO radar. Sensors.

[9] Abeida H., Delmas J. (2006). MUSIC-like estimation of direction of arrival for noncircular sources. IEEE Trans. Signal Process..

[10] Zoubir A., Chargé P., Wang Y. Non circular sources localization with ESPRIT. Proceedings of the European Conference on Wireless Technology (ECWT 2003).

[11] Haardt M., Romer F. Enhancements of unitary ESPRIT for non-circular sources. Proceedings of the
2004 IEEE International Conference on Acoustics, Speech, and Signal Processing.

[12] Xu Y., Liu Z. (2009). Modified virtual spatial smoothing algorithm. Acta Electron. Sin..

[13] Liu J., Huang Z., Zhou Y. (2008). Extended 2q-MUSIC algorithm for noncircular signals. Signal Process..

[14] Krim H., Viberg M. (1996). Two decades of array signal processing research: the parametric approach. IEEE Signal Process. Mag..

[15] Liu J., Zhang Y., Lu Y., Wang W. (2017). DOA estimation based on multi-resolution difference co-array perspective. Digit. Signal Process..

[16] Wang X., Chen Z., Ren S., Cao S. (2017). DOA estimation based on the difference and sum coarray for coprime arrays. Digit. Signal Process..

[17] Qin S., Zhang Y.D., Amin M.G. (2017). DOA estimation of mixed coherent and uncorrelated targets exploiting coprime MIMO radar. Digit. Signal Process..

[18] Sun F., Wu Q., Sun Y., Ding G., Lan P. (2017). An iterative approach for sparse direction-of-arrival estimation in co-prime arrays with off-grid targets. Digit. Signal Process..

[19] Harry L., Trees V. (2002). Optimum Array Processing: Part IV of Detection, Estimation, and Modulation Theory.

[20] Moffet A. (1968). Minimum-redundancy linear arrays. IEEE Trans. Antennas Propag..

[21] Pal P., Vaidyanathan P. (2010). Nested arrays: A novel approach to array processing with enhanced degrees of freedom. IEEE Trans. Signal Process..

[22] Vaidyanathan P., Pal P. (2011). Sparse sensing with co-prime samplers and arrays. IEEE Trans. Signal Process..

[23] Liu C.L., Vaidyanathan P.P. (2016). Super nested arrays: Linear sparse arrays with reduced mutual coupling–Part I: Fundamentals. IEEE Trans. Signal Process..

[24] Qin S., Zhang Y., Amin M. (2015). Generalized coprime array configurations for direction-of-arrival estimation. IEEE Trans. Signal Process..

[25] Abramovich Y.I., Gray D.A., Gorokhov A.Y., Spencer N.K. (1998). Positive-definite toeplitz completion in DOA estimation for nonuniform linear antenna arrays. I. Fully augmentable arrays. IEEE Trans. Signal Process..

[26] Abramovich Y.I., Spencer N.K., Gorokhov A.Y. (1999). Positive-definite toeplitz completion in DOA estimation for nonuniform linear antenna arrays. II. Partially augmentable arrays. IEEE Trans. Signal Process..

[27] Pillai S., Haber F. (1987). Statistical analysis of a high resolution spatial spectrum estimator utilizing an augmented covariance matrix. IEEE Trans. Acoust. Speech Signal Process..

[28] Liu C.L., Vaidyanathan P.P., Pal P. Coprime coarray interpolation for DOA estimation via nuclear norm minimization. Proceedings of the 2016 IEEE International Symposium on Circuits and Systems (ISCAS).

[29] Pal P., Vaidyanathan P. Coprime sampling and the MUSIC algorithm. Proceedings of the 2011 IEEE Digital Signal Processing Workshop and IEEE Signal Processing Education Workshop (DSP/SPE).

[30] Ren S., Wang W., Chen Z. DOA estimation exploiting unified coprime array with multiperiod subarrays. Proceedings of the 2016 CIE International Conference on Radar (RADAR).

[31] Wang W., Ren S., Chen Z. (2018). Unified coprime array with multiperiod subarrays for direction-of-arrival estimation. Digit. Signal Process..

[32] Ma W.K., Hsieh T.H., Chi C.Y. (2010). DOA estimation of quasi-stationary signals with less sensors than sources and unknown spatial noise covariance: a Khatri–Rao subspace approach. IEEE Trans. Signal Process..

[33] Shen Q., Liu W., Cui W., Wu S. (2016). Extension of co-prime arrays based on the fourth-order difference co-array concept. IEEE Signal Process. Lett..

[34] Wan L., Xie L. An improved DOA estimation algorithm for circular and non-circular signals with high resolution. Proceedings of the 2016 IEEE International Conference on Acoustics, Speech and Signal Processing (ICASSP).

[35] Gao F., Nallanathan A., Wang Y. (2008). Improved MUSIC under the coexistence of both circular and noncircular sources. IEEE Trans. Signal Process..

[36] Chen H., Hou C., Liu W., Zhu W.P., Swamy M.N.S. (2016). Efficient two-dimensional direction-of-arrival estimation for a mixture of circular and noncircular sources. IEEE Sens. J..

[37] Chen Z., Ren S., Wang W. DOA estimation exploiting extended co-array of coprime array. Proceedings of the 2016 CIE International Conference on Radar (RADAR).

